# Differentiating atomoxetine-induced agitation from mania relapse in a patient with bipolar disorder and ADHD: a case report

**DOI:** 10.1139/jpn-25-0144

**Published:** 2026-02-23

**Authors:** Vandad Sharifi, Valerie Taylor

**Affiliations:** ^a^ Department of Psychiatry, 2129 University of Calgary, Calgary, Canada

A 29-year-old woman with Bipolar I Disorder, stable for years on lithium 750 mg and quetiapine 250 mg, sought treatment for a confirmed ADHD diagnosis, with onset in early childhood. Medical history was otherwise unremarkable, noting only idiopathic intracranial hypertension in remission, and no prior ADHD medication or other significant health or substance use concerns. No previous manic episodes were reported in association with antidepressant trials.

Upon initiating atomoxetine 40 mg, she rapidly developed severe physical sensations over her eyes, arms, and legs (“buzzing under the skin”), and intense restlessness that worsened with rest. She reported trouble falling and staying asleep, but not a decreased need for sleep. She also experienced increased energy, racing thoughts, irritability, talkativeness, and pressured speech, similar to previous milder manic relapses, but without other typical manic symptoms like elevated mood, overspending and hypersexuality. While she had experienced milder physical sensations during past relapses, these were far more severe. Physical exam and labs were normal, and her recent lithium level was 0.49 mmol/L.

Atomoxetine was discontinued after 2 weeks. We briefly increased her lithium to 900 mg, then returned it to 750 mg due to sedation. Symptoms fully resolved 2 weeks post-discontinuation, and she remained stable over a 3-month follow-up.

This report describes a stable bipolar patient who developed severe physical sensations, restlessness, and emotional dysregulation on atomoxetine prescribed for ADHD, underscoring the diagnostic challenge of differentiating medication side effects from a manic relapse.

ADHD is a common comorbidity in adults with bipolar disorder, with a pooled prevalence of 18.6% according to a recent meta-analysis,^1^ often necessitating pharmacotherapy. However, managing ADHD in this population presents significant challenges. While stimulants and atomoxetine (a selective norepinephrine reuptake inhibitor) may be considered as add-ons to mood-stabilizing treatment in stable patients, robust safety evidence safety remains limited.^2^ An inherent risk of manic relapse exists, and it is often difficult to differentiate a true relapse from medication-induced side effects like restlessness or irritability. Caution is warranted as most ADHD treatment trials exclude patients with bipolar disorder.^2^

In our patient, agitation and discomforting physical sensations were the most prominent symptoms. While restlessness is a less common side effect of atomoxetine,^3^^,^^4^ some case studies describe akathisia-like presentations.^5^^,^^6^ Akathisia, characterized by an inner sense of restlessness and an irresistible urge to move, strongly aligns with our patient's subjective experience. However, she also reported symptoms resembling previous manic relapses, such as racing thoughts and pressured speech, indicating that akathisia alone could not fully explain her reaction.

The patient's presentation shared similarities with Activation Syndrome and Restless Legs Syndrome (RLS). Activation Syndrome, more commonly seen with antidepressants, involves hyperarousal, agitation, insomnia, and irritability.^7^ Yet, our patient's unique severe internal “buzzing” and emotional disturbances, reminiscent of past manic episodes, differentiated her case from a typical activation syndrome. RLS, characterized by an urge to move the legs with or without sensations, was also unlikely. Her restlessness and physical sensations were not primarily in her legs and were coupled with emotional dysregulation. Notably, atomoxetine generally improves, rather than causes, RLS symptoms.^8^

A key concern when treating ADHD in bipolar patients is the risk of manic relapse. While some case reports link atomoxetine to hypomania/mania, published studies generally indicate a low risk of manic switch, especially in adults.^9–13^ However, most trials exclude bipolar patients, which limit robust safety data. Furthermore, the current level of evidence is too low for confident risk quantification.

Our patient's increased irritability, racing thoughts, pressured speech, and heightened energy mirrored past manic relapses, leading to a lithium dose increase. Interestingly, she had also experienced milder “buzzing” sensations in prior relapses. Although it would be difficult to clearly differentiate a manic relapse from an atomoxetine side effect in this patient, we suspect the clinical picture reflects a mixture of both. It is plausible that atomoxetine exacerbated the dopaminergic and noradrenergic hyperactivity associated with acute mania,^14^^,^^15^ contributing to both her prominent akathisia-like restlessness and manic symptoms resembling previous episodes.

This case underscores the critical need for clinicians to closely monitor bipolar patients initiating atomoxetine for ADHD. Careful risk-benefit assessment is essential, as is the ability to differentiate between a true manic switch and treatment-related side effects. Prompt discontinuation of ADHD medication and adjusting bipolar treatment may be crucial.
